# Growth Inhibition by Bupivacaine Is Associated with Inactivation of Ribosomal Protein S6 Kinase 1

**DOI:** 10.1155/2014/831845

**Published:** 2014-01-29

**Authors:** Mushtaq Ahmad Beigh, Mehvish Showkat, Basharat Bashir, Asma Bashir, Mahboob ul Hussain, Khurshid Iqbal Andrabi

**Affiliations:** ^1^Department of Biotechnology, Science Block, University of Kashmir, Srinagar J&K 190006, India; ^2^Department of Biotechnology and Bioinformatics, University of Kashmir, Srinagar J&K 190006, India

## Abstract

Bupivacaine is an amide type long acting local anesthetic used for epidural anesthesia and nerve blockade in patients. Use of bupivacaine is associated with severe cytotoxicity and apoptosis along with inhibition of cell growth and proliferation. Although inhibition of Erk, Akt, and AMPK seemingly appears to mediate some of the bupivacaine effects, potential downstream targets that mediate its effect remain unknown. S6 kinase 1 is a common downstream effector of several growth regulatory pathways involved in cell growth and proliferation known to be affected by bupivacaine. We have accordingly attempted to relate the growth inhibitory effects of bupivacaine with the status of S6K1 activity and we present evidence that decrease in cell growth and proliferation by bupivacaine is mediated through inactivation of S6 kinase 1 in a concentration and time dependent manner. We also show that ectopic expression of constitutively active S6 kinase 1 imparts substantial protection from bupivacaine induced cytotoxicity. Inactivation of S6K1 though associated with loss of putative mTOR mediated phosphorylation did not correspond with loss of similar phosphorylations in 4EBP1 indicating that S6K1 inhibition was not mediated through inactivation of mTORC1 signaling pathway or its down regulation.

## 1. Introduction

Bupivacaine is an amide type local anaesthetic used in clinical pain management [1]. Although considered safe, considerable side effects associated with its use remain a major concern. Bupivacaine though variable in efficacy inflicts myotoxicity and neurotoxicity and is also implicated in slowing down or preventing wound healing at surgical sites [2–6]. These effects have largely been attributed to its influence on cellular proliferation and cell death due to necrosis or apoptosis [7–9]. Accordingly studies have suggested that bupivacaine induced cell damage may involve participation of mitogen activated protein kinase (MAPK) and protein kinase B (Akt) signaling pathways [10–12]. Similarly disruption of other signalling events has been suggested for myotoxic effects associated with its use [13, 14]. Since most of the pathways converge on S6 kinase, it was imperative to examine its relevance in mediating growth inhibitory effects associated with the drug.

Ribosomal protein S6 kinase 1 (S6K1) is an evolutionary conserved protein kinase that acts downstream of mTOR/PI3-kinase/Akt signalling pathway [15–18]. Growth factor dependent activation of this kinase is essential for the cell cycle progression primarily due to its influence on protein synthesis [19]. In addition, the role of S6K1 stands established in other cellular processes like autophagy, apoptosis, and aging implicating a complex network of signalling events in its regulation [20–22]. mTORC1 complex is considered to be the major activating input that regulates cellular growth through downstream effectors S6K1 and 4EBP1 (eukaryotic initiation factor 4E binding protein 1) [23]. Interaction between MAPK and S6K1 suggestive of cross talk between these two pathways stands well characterized, such that MAPK/Erk inhibitor, namely, PD-98059, inactivate S6K1 [24]. A recent observation by Choi et al. indicates that cellular death and survival pathways are regulated by Erk/GSK3*β*/S6K1 axis [25]. Similarly Akt—a downstream effector of PI3-Kinase pathway is a known activator of S6K1 that caters to the response associated with insulin signalling [26].

Since the effects associated with bupivacaine are at least in part believed to be mediated by signals with potential to modulate S6K activity, we attempted to investigate such a possibility for further insights into the antiproliferative effects of the anaesthetic and relate it with inhibition of S6K pathway. Long established inhibitory influence of bupivacaine on amino acid sensing through tRNA charging invites further interest in potential involvement of S6 kinase pathway known to be critical for maintaining cellular nutrient homeostasis [27, 28]. Inhibition of such signals is therefore likely to have a bearing on the activity status of the S6K1 enzyme.

## 2. Materials and Methods

### 2.1. Materials and Antibodies

Bupivacaine (Astra Zeneca), Protein G-Agarose beads (Calbiochem), DMEM, FBS, and antibiotics were purchased from Sigma-Aldrich; all other reagents were purchased from Sigma unless otherwise indicated. Anti-S6K antibody (Santa Cruz Biotechnologies, Inc.), Anti-S6K (Polyclonal-Genscript), Anti-Erk1, Anti-phospho-Erk, Anti-4EBP1, Anti-phospho-4EBP1-T70 and Anti-phospho-S6K-T412 antibodies were obtained from Cell Signaling Technologies, Anti-phospho-S6K-T252 antibody was from R&D systems, and Anti-PARP antibody was from Abcam, Inc. USA. PVDF membrane was from Millipore, Inc. and, goat anti-rabbit secondary antibody conjugated to IR Dye 800CW was from LI-COR Biotechnologies.

### 2.2. Plasmid Constructs

HA (hemagglutinin) tagged S6 kinase, constitutively active HA-S6 kinase (S6K-T412E-DE3) and GST-S6 used as a substrate in S6K kinase assays were generated as described previously [29, 30].

### 2.3. Cell Culture and Transfections

NIH-3T3 fibroblasts, C2C12 myoblasts and C6 neuroglial cells were cultured in Dulbecco's modified Eagle's medium (with appropriate antibiotics) containing 10% (v/v) fetal bovine serum in an atmosphere of 5% CO_2_. Transfections were carried out using lipofectamine reagent (Invitrogen, Inc.) as per manufacturer's instructions in a 6-well plate format.

### 2.4. Drug Treatment, Immunoprecipitation, and Western Blotting

NIH-3T3 cells were incubated with indicated concentrations of drugs overnight or as shown. Cells were lysed in a buffer containing 50 mM Tris-HCl (pH 7.5), 10 mM MgCl_2_, 5 mM EDTA, 2 mM DTT, 50 mM *β*-glycerophosphate, 0.5% Triton X-100 and protease inhibitor cocktail (Sigma) for 30 minutes and centrifuged at 15000 g for 30 minutes to clear the lysate. Clarified lysates were incubated with Anti-S6K antibody (mouse monoclonal immobilized on protein G-Agarose beads) for 2–4 hrs. Beads were washed thrice with lysis buffer containing 500 mM NaCl before final washings with kinase buffer.

### 2.5. Immune Complex Kinase Assays

S6K1 immunoprecipitates were incubated with 1 µg GST-S6 and 5 µCi p^32^-ATP in a kinase reaction buffer containing 50 mM Tris-Cl (pH 7.0), 10 mM MgCl_2_, 0.5 mM DTT, 50 mM *β*-glycerophosphate, and 1 mM ATP for 20 minutes at 37°C. Reaction was stopped by adding 2 X loading buffer and separated on a 12% SDS-PAGE gel. Proteins were transferred on PVDF membrane, autoradiographed, and probed with S6K antibody (rabbit polyclonal) and other phospho antibodies followed by analysis with ODYSSEY infrared imager (LI-COR).

### 2.6. Proliferation Assays

Cells in triplicate (plated at density of 5000 cells per well) were grown in growth media in the presence or absence of bupivacaine for 24 hrs or as indicated in 96-well format and subjected to MTT assays. The yellow tetrazolium salt MTT was reduced to formazan by intracellular NADPH-oxidoreductases. The formazan crystals were solubilized and quantified by spectrophotometry using plate reader (Epoch Biotech, USA). Assays were performed using an MTT cell proliferation kit (Sigma Aldrich, USA).

### 2.7. In-Cell Western Assays and Fluorescence Quantitation

In-cell western assays were done according to the protocol provided by LI-COR Biotechnologies. Briefly, NIH-3T3 cells seeded in 12-well plates were treated with S6K1 inhibitory concentrations of bupivacaine overnight. For analysis, cells were washed with PBS, fixed using 4% paraformaldehyde, permeabilized using 0.1% Triton X-100 in PBS, blocked, and then incubated with Anti-phospho-S6K antibodies in LI-COR blocking reagent overnight and average florescence intensity (in arbitrary units) of each well was quantitated using ODYSSEY infrared imager (LI-COR Biotechnologies).

### 2.8. Cell Death Assay

ELISA based cell death detection kit (Roche Applied Sciences) was used to detect apoptosis after bupivacaine treatment. The assay is based on a quantitative sandwich ELISA using antibodies directed against DNA and histones to detect mono- and oligonucleosomes in the cytoplasm of cells undergoing apoptosis. ELISA was carried out according to the manufacturer's protocol. Measured OD was normalized for cell number (protein content).

### 2.9. Statistical Analysis and Calculation of IC50

Representative experiments from three independent experiments are shown. Results for each experiment are given as mean of triplicates ± SD. Statistically significant differences between samples are determined using Student's t-tests (Excel, Microsoft). The half maximal inhibitory concentration (IC50) of bupivacaine was determined by nonlinear least-squares fitting of the data of MTT assays using the following equation: Normalized Cell Number = 1/(1 + [BPV]/IC50), with Graph Pad Prism Software (Version 4.0).

## 3. Results

### 3.1. Bupivacaine Induces Toxicity in a Cell Type Specific and Dose-Dependent Manner

**Figure 1:**
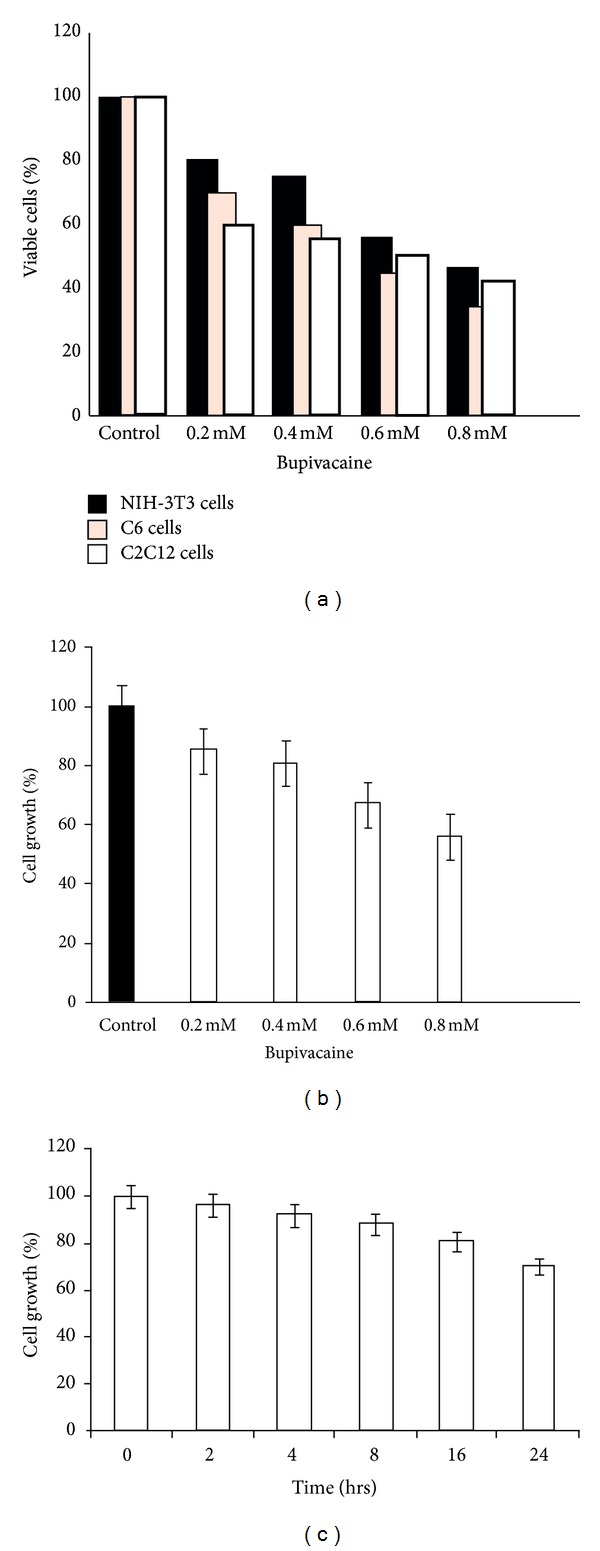
Dose-dependent inhibition of cell growth. (a) NIH-3T3, C2C12, and C6 neuroglial cells were plated at a cell density of 5 × 10^3^cells /well in triplicate under standard growth conditions (96-well format) for 24 hrs and incubated with indicated concentrations of bupivacaine. Cells were washed with cold PBS and subjected to MTT assays. (b) Dose-dependent inhibition of cell growth in NIH-3T3 cells exposed to bupivacaine at indicated concentrations for 24 hrs. (c) Time dependent inhibition of cell growth in NIH cells treated with bupivacaine (0.614 mM). Cell growth rate was normalized to the control. Data are mean ± SEM (*n* = 3). *P* < 0.05 versus control.

Cytotoxicity of bupivacaine was evaluated by its effect on cell viability. Accordingly cell lines NIH-3T3, C6, and C2C12 myoblasts were allowed to grow in the absence (control) or presence of bupivacaine at various concentrations over a period of 24 hrs. While the control cells exhibited normal growth characteristics, significant dose-dependent decline in cellular density was observed in bupivacaine treated cells resulting in substantial cell death (Figure 1(a)). The magnitude of cell death, however, varied with individual cell type such that maximum sensitivity to bupivacaine induced toxicity was most pronounced for C6 neurological cells recording more than 60% death at concentrations (0.2 mM) which did not cause substantial decline in densities of other cell types. While an intermediate toxicity response was observed for C2C12 cells, the NIH-3T3 cells were the least sensitive with an IC50 of 0.614 mM which were accordingly deemed appropriate for reliable assessment of signaling dynamics associated with bupivacaine induced toxicity without undue interference from cessation of cellular processes. NIH-3T3 cells were used further for studying their behavior in response to bupivacaine treatment and loss of cell viability potentiated by morphological changes like cell rounding and detachment was associated with a dose-dependent decrease in cellular proliferation as assessed by MTT assay (Figure 1(b)). A time dependent assay in NIH cells with minimal inhibitory concentration (0.614 mM) showed cells to be more than 70% viable at 24 hrs (Figure 1(c)).

### 3.2. Bupivacaine Inhibits S6K1 in a Concentration and Time Dependent Manner

**Figure 2:**
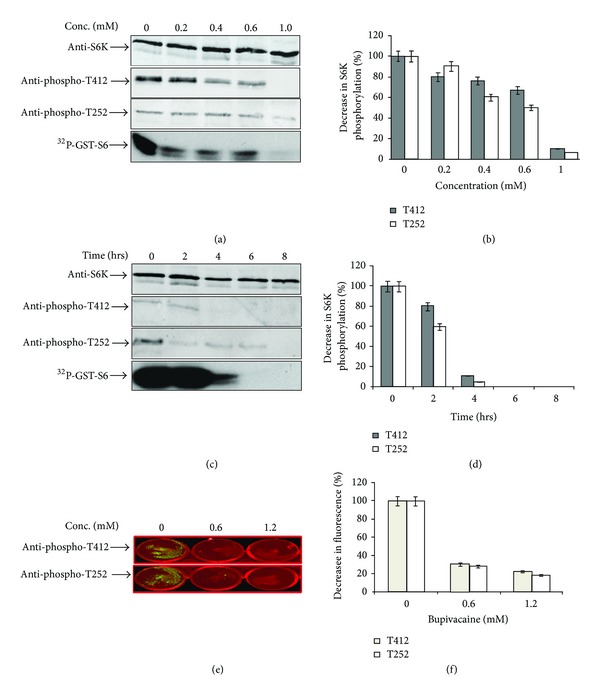
Concentration and time dependence of S6K1 inhibition by bupivacaine. (a) NIH-3T3 cells were allowed to grow for 12 hrs in the absence (control) or presence of indicated concentrations of bupivacaine (BPV) and S6K1 was immunoprecipitated, subjected to kinase assays, and then probed with indicated antibodies. (b) Densitometric analysis of anti-pS6K signals (normalized to total S6K levels) from three independent experiments. Data are relative S6K phosphorylation levels with control set as 100%, presented as mean ± SEM. (c) NIH-3T3 cells were allowed to grow in the absence or presence of bupivacaine (1 mM) for indicated time intervals and processed similarly as above. (d) Densitometric analysis of anti-pS6K signals (normalised to total S6K levels) from three independent experiments. (e) NIH-3T3 cells were incubated with inhibitory concentrations (1 mM) of bupivacaine as described above. Cells were processed for treatment with S6K Phospho-T412 and S6K Phospho-T252 antibodies and imaged using LI-COR infrared imager. (f) Average florescent intensity of each well was calculated in arbitrary units (AU) using LI-COR ODYSSEY software.

Growth inhibition and apoptosis have quite often been associated with dysregulation of signaling pathways with potential to influence S6K1 activity directly or indirectly. We therefore, sought to investigate any such possibility by analyzing activity status of S6K1 in the presence or the absence of the drug. Endogenous S6K1 was immunoprecipitated from NIH-3T3 cells grown in presence or absence of different bupivacaine concentrations for its ability to phosphorylate GST-S6. As seen in Figure 2(a), bupivacaine caused S6K1 inhibition in a concentration dependent manner with its near complete inhibition at a drug concentration of 1 ± 0.06 mM. Minimal inhibitory concentration for S6K1 was then used to establish the time course of S6K1 inhibition. Figure 2(c) shows that inhibition of S6K1 activity was stringently time dependent with more than 80% inhibition observed at 4 hours of the drug exposure. The inhibitory time course was in concordance with inhibition of cell proliferation.

### 3.3. S6K1 Inhibition Is Associated with Loss of Activating Phosphorylations

Catalytic and linker domain phosphorylations at the activation loop (AL) and hydrophobic motifs (HM) are established determinants of S6K1 enzyme activity. Accordingly their loss is a hallmark of S6K1 inhibition. We therefore sought to ascertain whether inhibition of S6K1 by bupivacaine did indeed correspond with loss of these phosphorylations. As seen in Figure 2(a), both T412 and T252 phosphorylations were lost in a concentration dependent manner which corresponded with activity profile of the enzyme to exhibit a 20–90% drop in fluorescent intensity over the entire range of drug concentrations tested (Figure 2(b)). The loss of both the phosphorylations followed a strict time course with 90% loss of both the phosphorylations at 4 h (Figure 2(d)). In order to rule out that the observed loss of S6K1 phosphorylations was not due to a contaminating phosphatase, an in-cell western assay in NIH-3T3 cells using phospho specific antibodies against Threonine 412 and Threonine 252 in S6 kinase was performed in the presence or absence of the drug. As seen in Figure 2(e), the relative fluorescent intensity for the individual phospho-antibodies exhibited similar pattern of disappearance as observed for the immunoprecipitated enzyme. At bupivacaine concentration of 1.2 mM the relative phosphorylation decreased to 20% when quantitated using LI-COR infrared imager (Figure 2(f)).

### 3.4. Constitutive Active S6K1 Induces Resistance to Bupivacaine Toxicity

**Figure 3:**
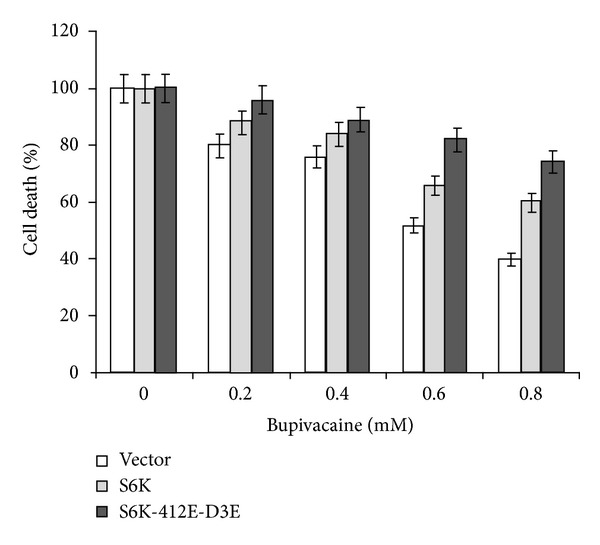
Constitutively active S6 kinase prevents cells from bupivacaine induced toxicity: NIH-3T3 cells transfected with HA-S6K1 and constitutively active S6K (S6K-T412E-D3E). 48 hrs posttransfection cells were exposed to various concentrations of bupivacaine (1 mM) for 12 hrs before harvest. Data are representative of three independent experiments.

In an attempt to establish that S6K1 inactivation did indeed mediate the growth inhibitory effects associated with bupivacaine, cells transfected with HA-tagged S6K1 wild type and a constitutively active variant T412E-D3E were examined for their ability to resist bupivacaine induced toxicity.  Figure 3 shows that while WT S6K1 was able to significantly improve the live cell number, it remained partially protective (30%) in comparison to a dramatic 80–85% protection conferred in cells expressing the constitutive active variant of the enzyme. These data clearly indicate that S6K1 did indeed mediate growth inhibition by bupivacaine.

### 3.5. S6K1 Inhibition by Bupivacaine Is mTORC1 Independent

**Figure 4:**
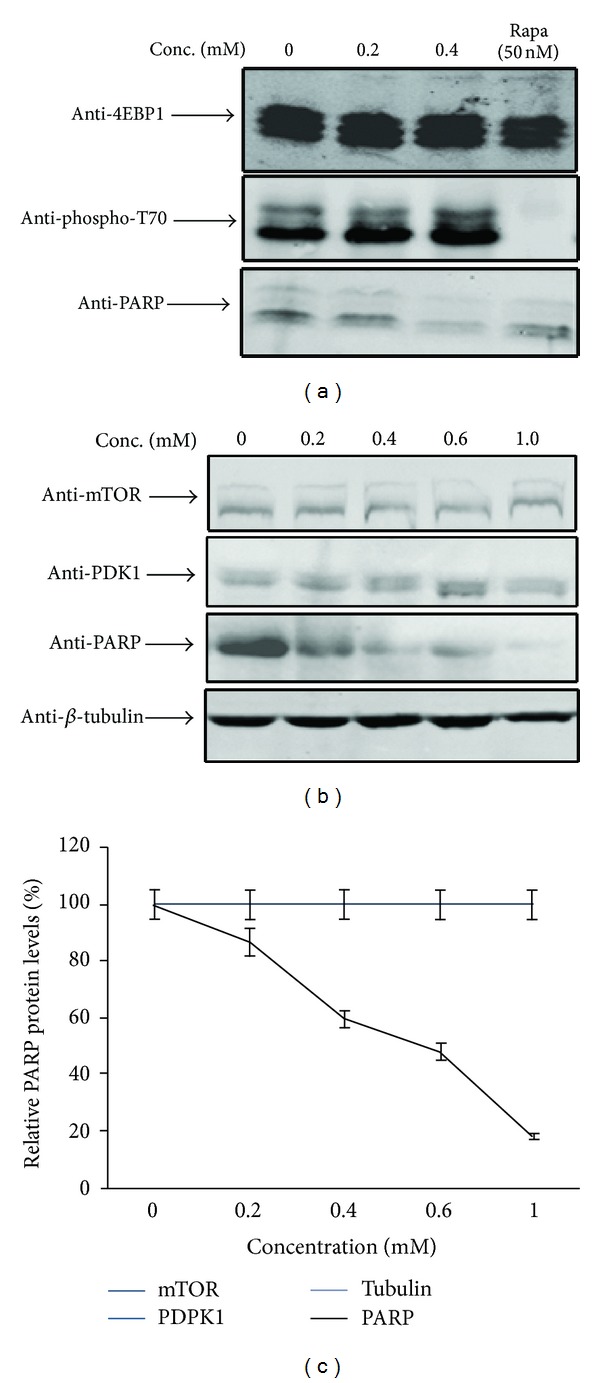
BPV mediated inhibition is independent of mTOR input: (a) NIH-3T3 cells were exposed to various concentrations of bupivacaine and incubated for 12 hrs before harvest. 4EBP1 was immunoprecipitated with monoclonal antibody cross-linked to protein G-Agarose beads and immune complexes recovered were separated on a 15% SDS-PAGE gel, transferred on PVDF membrane, and immunoblotted with rabbit anti-4EBP1 and anti-phospho-T70; Rapamycin was used as a positive control to inhibit mTORC1 complex (lane 4). (b) NIH-3T3 cells were grown in the absence (control) or presence of bupivacaine (1 mM) for 12 h and subjected to immunoblotting with anti-mTOR, anti-PDPK1, anti-PARP, and anti *β*-tubulin antibodies, respectively, to detect endogenous levels of these proteins. (c) Expression analysis of *β*-tubulin relative to PARP protein levels (positive control) using fluorescence based quantitation by LI-COR-ODYSSEY software. Data are mean of three independent experiments.

mTORC1 is considered to be a major input acting upstream of S6K1 and implicated in T412 phosphorylation of the enzyme and parallel phosphorylations of its other substrate, eukaryotic translation initiation factor 4E binding protein 1 (4EBP1). Possible inactivation of mTOR pathway suggested by inhibition of S6K1 should therefore be corroborated by its inability to cause phosphorylations in 4EBP1 (Threonine 70). Phosphorylation of 4EBP1 at Threonine 70 site (Figure 4(a)) remained unchanged in the presence of bupivacaine when compared to its inhibition by specific mTORC1 inhibitor rapamycin. To completely rule out any possible mTORC1 input for S6K inhibition mediated by bupivacaine, an immunoblot analysis of endogenous mTOR and PDPK1 (Figure 4(b)) indicated that the drug did not cause any significant change in the expression of either kinase implicated in phosphorylation at HM (T412) and AL (T252), respectively, when compared to PARP (poly-ADP ribose polymerase) that recorded visible decrease in protein levels (Figure 4(c)) in conformity with its reported response to bupivacaine [31]. This goes to suggest that the selective inhibition of S6K1 was in no way related to change in activity or expression characteristics of mTOR or PDPK1.

### 3.6. S6K1 Inhibition by Bupivacaine Overlaps with Erk Inactivation

**Figure 5:**
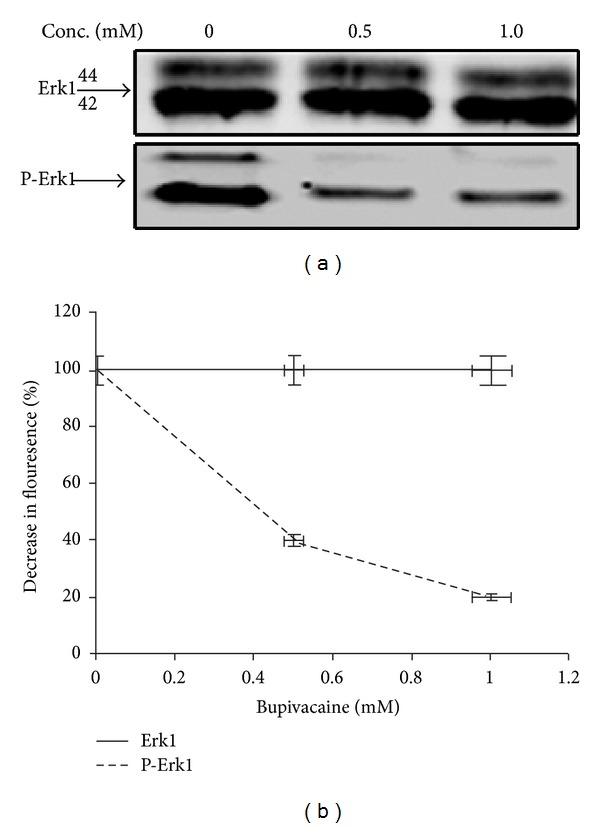
Overlapping time course of inhibition between Erk and S6K1 indicates bupivacaine as specific inhibitor of Erk and S6K1: (a) NIH-3T3 cells treated with indicated concentrations of bupivacaine as above. Lysates were clarified, immunoprecipitated, and probed with indicated antibodies. (b) Densitometric analysis of anti-P-Erk1 signals (normalized to total Erk levels) from three independent experiments. Data are relative Erk activation levels with control set as 100%, presented as mean ± SEM.

In the absence of mTOR as the mediator of S6K1 inhibition by bupivacaine, it was imperative to examine other signaling pathways that may directly or indirectly influence S6K1 activity in the cell. Since extracellular signal-related kinase/s (Erk) are believed to participate in the events leading to cytotoxicity by anesthetic drugs like bupivacaine, we sought to investigate the activation status of Erks to possibly relate it with observed inhibition of cellular proliferation and S6K1 activity. The activation of Erks was evaluated by immunoblotting with a phospho specific antibody. As seen in Figure 5(a), bupivacaine caused a dramatic reduction in Erk phosphorylation in a dose-dependent manner. Densitometric analysis (Figure 5(b)) of the data from several immunoblots (*n* = 3) indicated that the extent of Erk inhibition coincided with the inhibition of S6K1 as much as it did with the kinetics of cell proliferation, to suggest that the effect of bupivacaine may be mediated through inactivation of Erk and S6K1.

## 4. Discussion

The use of local anesthetics like bupivacaine is associated with chondrotoxicity, myotoxicity, and neurotoxicity at variable propensities [4, 9, 32]. Diverse spectrums of cellular changes that underline dysfunctional signal transduction are attributed to mediate cellular damage associated with their use [32]. Inhibition of cell growth, a key effect associated with the use of these drugs particularly bupivacaine, has invited significant interest leading to the identification of a series of signaling molecules that appear to mediate the process [11, 32]. Since growth regulatory and apoptotic mechanisms are largely interdependent and complexed further by cross talk among signaling pathways, the attribution of one or the other event to mediate such effects may be premature. Further, the influence of the other signaling pathways like S6K1 on cellular growth and apoptosis is as pronounced as, if not more than, the events reported to get influenced by such drugs [8, 10, 20]. Incidentally the effects associated with bupivacaine that include metabolic stress, influence on nutrient homeostasis, and apoptosis directly or indirectly suggest a possible involvement of S6K pathway, to rationalize this study.

We chose NIH-3T3 cells for their restrained response to bupivacaine induced cytotoxicity compared to C2C12 and C6 cell lines, to help us register the dynamics of signaling events responsible for preventing cell growth and proliferation. We demonstrate that the inhibition of cell proliferation by bupivacaine strictly correlated with the inactivation of ribosomal protein S6 kinase 1 and its relief by the use of constitutively active enzyme to suggest a possible dysregulation of events that control cell division, at least in part via protein translation. With long standing implications that bupivacaine may influence protein synthesis by interfering with amino acylation of tRNAs [28], the relevance of S6K1 inhibition becomes even more compelling. Therefore, evaluating the status of different signaling molecules known to influence S6K1 activity directly or indirectly becomes imperative to establish their relation with bupivacaine action. The principal regulatory input for S6K1 is believed to be provided by mTOR signaling pathway wherein mTOR kinase directly phosphorylates S6K1 at its hydrophobic motif to trigger a second phosphorylation at its activation loop for full activation [27, 33]. Sequential dynamics of the two phosphorylations is, therefore, an established index of S6K1 activity and often related to the activity status of the mTOR pathway per se. However, DRAK2, Akt, GSK3, and Erk like kinases believed to phosphorylate putative mTOR site in S6K1 rope in other signaling pathways as possible regulators of S6K1 [17, 24, 34, 35]. Bupivacaine induced loss of S6K1 phosphorylation at putative mTOR site and at the activation loop as a consequence, without any perceptible change in mTOR kinase activity or expression observed herein, substantiates the later possibility even further. Incidentally, all these kinases have established influence in the process of cell growth and apoptosis and each would easily fit into a role of mediating growth inhibition by bupivacaine, with a marginal preference at best, for one over the other. Despite its established role in cellular apoptosis, DRAK2 may slip down the preference list for its immune cell specific response, yet its participation in the mechanistic events associated with bupivacaine cannot be ruled out completely. On the other hand, Akt presents credible candidature in light of its established role in S6K regulation coupled with its documented inhibition by bupivacaine [11, 26]. Since Akt influence on S6K1 remains largely dependent on its cross talk with mTOR pathway [36], its exclusive participation in mediating growth inhibition by bupivacaine becomes difficult to explain. That is perhaps why Akt has often been implicated in mediating apoptosis, an event that comes next to growth inhibition. Our observation that Akt phosphorylation by bupivacaine was inhibited much later in time and at concentrations higher than required for S6K inhibition (data not shown) may support the contention further. However, involvement of other signaling pathways that may recruit Akt to mediate other effects associated with bupivacaine cannot be ruled out. This is particularly relevant in the context of the data that p38MAPK and c-Jun N-terminal kinase pathways stand implicated in bupivacaine induced neurotoxicity [10, 11, 32], whereas mitochondrial dysfunction and altered calcium homeostasis are suggested to mediate myotoxicity [14, 37]. These observations indicate the complex nature of events that may collaborate to bring about cumulative effects associated with bupivacaine or else the drug may engage distinct signaling pathways in a function specific and/or a cell specific manner.

MAPK signaling has a central role in regulating cell growth and proliferation [38]. Accordingly cell division is facilitated by activation of Erks and prevented by agents with the ability to inhibit their activation [39, 40]. The contribution of S6K1 in promoting cell division is no less pronounced and equates well with that of Erks especially in the context of its inhibition by bupivacaine with overlapping dose and time kinetics. The observation lends support to the contention of a cross talk between Erk and S6K1 pathways, in agreement with the data describing MAPK-S6K1 coimmunoprecipitation to suggest a coordinated Erk-GSK3B-S6K1 axis for cell growth and proliferation [12, 24, 34]. Incidentally both Erk and GSK3 are known to influence phosphorylation status of S6K1 to contribute in direct activation of S6K1 [34]. Accordingly agents that inhibit upstream members of the MAPK pathway resulting in ERK inactivation also seem to influence the activity status of S6K1. It is, however, premature to attribute a general role for Erks in S6K1 activation in preference to mTOR and it should instead be considered a consequence associated with the use of bupivacaine. Paradoxically, the contention that mTOR regulates S6K1 by a mechanism other than phosphorylation [30] may implicate Erks in a more general role.

## 5. Conclusions

Our data suggests that bupivacaine induced inhibition of cell proliferation is mediated at least in part through inactivation of S6 kinase 1. We also demonstrate redundant role of mTORC1 signaling in mediating this effect to suggest the possible involvement of Erk pathway.
